# The ADVanced Organ Support (ADVOS) hemodialysis system balances blood pH within 24 h in patients with multiple organ failure and hypercapnic acidosis

**DOI:** 10.1186/s40635-025-00820-1

**Published:** 2025-10-25

**Authors:** Miriam Dibos, Aritz Perez Ruiz de Garibay, Ulrich Mayr, Roland M. Schmid, Johannes Honigschnabel, Tobias Lahmer

**Affiliations:** 1 Klinik Und Poliklinik Für Innere Medizin II, Klinikum Rechts Der Isar der Technischen Universität München, Ismaninger Str. 22, 81675 Munich, Germany; 2 ADVITOS GmbH, Munich, Germany

**Keywords:** Hypercapnic acidosis, Extracorporeal CO2 removal, Albumin dialysis, ADVanced Organ Support, Acid–base balance

## Abstract

**Background:**

Severe hypercapnia, especially when associated with acidosis, should be avoided or actively managed, as it is an independent risk factor in critically ill patients. The ADVOS multi hemodialysis system offers the potential to correct acidosis and hypercapnia through customizable pH and bicarbonate content within the dialysate fluid. The aim of this work is to analyze the exact timing for pH correction and the main factors leading to it in patients with multiple organ failure (MOF) and hypercapnic acidosis treated with ADVOS.

**Methods:**

Patients with MOF and metabolic or hypercapnic acidosis (pH < 7.35) were included over a study period of 13 months. All patients received at least one treatment with ADVOS hemodialysis system for at least 24 h. The primary outcome was the time to blood pH ≥ 7.35.

**Results:**

24 patients with a median age of 61 years and a median SOFA score of 15 points were included. The results of 134 ADVOS sessions, with a median of 5 sessions per patient, were analyzed. Median time to blood pH ≥ 7.35 was 4 h; a significant blood pH increase within 24 h was reached in all patients (7.21 before vs. 7.39 after, *p* < 0.01).

**Conclusions:**

A single session of ADVOS corrected blood pH and supported the reduction of pCO_2_ with a median CO_2_ removal of 55 mL/min in patients with multiple organ failure and hypercapnic acidosis.

**Supplementary Information:**

The online version contains supplementary material available at 10.1186/s40635-025-00820-1.

## Background

Acidosis and hypercapnia are frequent complications in critically ill patients, often resulting from multiple organ failure. These disturbances disrupt acid–base homeostasis and are associated with increased mortality, prolonged ICU stays, and worsened clinical outcomes [1, 2]. Therefore, early and effective management is essential to mitigate their impact.

Hypercapnia typically arises from impaired carbon dioxide (CO_2_) elimination due to pulmonary failure, which can lead to secondary acidosis. In the following, elevated CO_2_ levels can trigger vasoconstriction in peripheral organs and vasodilation in cerebral vessels, contributing to increased intracranial pressure, but also impairing clinical outcomes by influencing cardiovascular response to therapy [3–5]. Current therapeutic strategies to manage pulmonary hypercapnia include non-invasive and invasive mechanical ventilation; however, if these modalities fail, therapeutic options are limited [6, 7]. Extracorporeal therapies such as extracorporeal carbon dioxide removal (ECCO_2_R) and extracorporeal membrane oxygenation (ECMO) can reduce CO_2_ levels directly through gas exchange membranes. However, these methods are resource-intensive, carry significant risks (e.g., bleeding and anticoagulation-related complications), and focus exclusively on respiratory support without addressing the broader metabolic derangements.

The ADVanced Organ Support (ADVOS) multi system, an albumin-based hemodialysis device, was initially developed for liver and kidney support in critically ill patients. Beyond its conventional use [8–13], the ADVOS multi system has demonstrated potential to correct acidosis and remove CO_2_ at low blood flows (i.e., 100–400 mL/min). This is possible through its pH-optimization capabilities, which include modulations of dialysate pH and bicarbonate content [14, 15]. Recent in vitro studies have highlighted the system’s ability to address hypercapnic acidosis effectively [16].

While promising, the ADVOS multi system’s clinical efficacy in rapidly correcting acidosis and CO_2_ levels in critically ill patients has not been systematically evaluated. Specifically, the time required to achieve acid–base correction in real-world settings and the influence of operational parameters—such as blood flow, concentrate flow, and dialysate composition—remain unexplored beyond controlled in vitro environments.

This study investigates the efficiency of the ADVOS system in correcting blood pH and eliminating CO_2_ in patients with MOF and hypercapnic acidosis. The primary aim is to determine the time required for pH normalization and identify the key factors influencing its effectiveness under varying operational conditions.

## Methods

### Study design, settings and patients

An observational study was conducted in a medical ICU at Klinikum Rechts der Isar in Munich, Germany. Between January 2021 and February 2022, all patients with MOF and hypercapnic acidosis hospitalized in the ICU were eligible for study inclusion. The inclusion criteria included patients with acute respiratory distress syndrome (ARDS) and acute kidney injury (AKI) either under mechanical ventilation for at least 72 h or with a contraindication to ECMO according to internal guidelines, who required urgent treatment for hypercapnic acidosis. Hypercapnic acidosis was defined by a blood pH below 7.35 in the presence of elevated arterial pCO₂ (> 45 mmHg) and normal or elevated plasma bicarbonate (HCO₃⁻ ≥ 21 mmol/L). This pattern reflects a primary respiratory origin of the acidemia, where CO₂ retention leads to decreased pH despite a compensatory or non-depleted bicarbonate level. Hypercapnic acidosis may be isolated or part of a mixed acid–base disorder if additional metabolic abnormalities are present.

To minimize selection bias, all eligible patients meeting the inclusion criteria were enrolled until a total of 24 patients was reached. All patients received one or more ADVOS multi treatment for 24 h. Primary outcome was time to blood pH ≥ 7.35.

### Ethics, consent and permissions

The present study was approved by the institutional review board of the Technical University of Munich, Germany 178/20S. Due to the retrospective nature of the study the requirement for written informed consent was waived. Data were pseudonymized before the analyses were performed. All procedures were conducted in accordance with institutional guidelines and with the ethical standards of the Declaration of Helsinki and its later amendments.

### ADVOS multi hemodialysis system

The ADVanced Organ Support (ADVOS) multi hemodialysis system (ADVITOS GmbH, Munich, Germany) is an albumin-based hemodialysis device designed to address acid–base imbalances, remove CO_2_, and eliminate water-soluble and protein-bound toxins [17]. It operates through three interconnected circuits: the extracorporeal circuit, the dialysate circuit, and the ADVOS multi circuit.

Briefly, toxins diffuse into the albumin-enriched dialysate through semipermeable membranes (ELISIO-19H, Nipro, Osaka, Japan). The dialysate’s albumin is regenerated in the ADVOS multi circuit via pH and temperature modulation, enabling continuous toxin removal. Acidic and alkaline concentrates are mixed to adjust the dialysate pH (7.2–10.0) during treatment. A higher basic-to-acidic concentrate ratio increases dialysate pH, raising sodium and lowering chloride levels, facilitating H^+^ removal and promoting acidosis correction.

The concentrate flow (160–320 mL/min) determines the volume of dialysate processed by convective transport in the ADVOS multi circuit, further optimizing acid and toxin removal. CO_2_ removal relies on a concentration gradient between blood and dialysate, with blood flows adjusted between 100 and 400 mL/min and dialysate flow set at 800 mL/min. In addition, the bicarbonate content in the dialysate can be adjusted using one of the three alkaline concentrates (BASE Bic 20, BASE Bic 10, BASE Bic 0), allowing either correction of metabolic acidosis (increased bicarbonate) or facilitation of CO_2_ removal (bicarbonate-reduced dialysate).

### Data assessment

Patients’ medical files were screened for age, sex, body mass index (BMI), medical history, and noradrenaline doses. ADVOS treatment parameters were filed using a clinical information system. For analysis, available parameters were evaluated immediately before and after each ADVOS treatment. Blood gas analyses were conducted at the inlet (pre-filter) and outlet (post-filter) of the dialyzer to assess the system’s capacity to remove CO_2_ and increase blood pH. CO_2_ removal (in mL/min) was calculated using the following equation:

$$V =({{\Delta \text{HCO}}_{3}}^{-}+ {\Delta p\text{CO}}_{2} *{K}_{\text{S}} ) *{Q}_{\text{b}} *{V}_{\text{m}}$$

Here, ΔHCO_3_^−^ and ΔCO_2_ represent the differences in bicarbonate (mmol/L) and CO_2_ partial pressure (mmHg) between the inlet and outlet of the dialyzer. *K*_S_​ is the solubility constant for CO_2_ in blood (0.03 mmol/mmHg), *Q*_b_ is the blood flow (L/min), and Vm is the molar volume of CO_2_ at standard temperature and pressure (22.4 mL/mmol).

The observation period extends until patient's discharge from the ICU or death, respectively. However, data from the first ADVOS treatment (i.e., 24 h) were only analyzed to assess the primary outcome of the study.

### Statistical analysis

Variables are presented as median values with the interquartile range (IQR), if not otherwise specified. The Shapiro–Wilk test was used to determine the data distribution. If not significant (i.e., normal distribution), differences between baseline and levels after the first 24-h treatment were calculated with the Student’s *t* test. The Wilcoxon signed rank test was employed for data without normal distribution. An ANOVA test with Bonferroni post hoc tests was applied to show significant differences between ADVOS treatment settings (i.e., blood flow, concentrate flow, dialysate pH and alkaline concentrate). In addition, to see if any correlation exists between ADVOS treatment settings and CO_2_ removal, first a bivariate analysis for Pearson correlation was done. Then a multiple linear logistic regression model was developed to evaluate the combined effects of the ADVOS settings on CO_2_ removal. Statistical significance was stated with a two-tailed *p* < 0.05. The cumulative percentage of patients reaching a blood pH > 7.35 is depicted through a Kaplan–Meier curve. IBM SPSS Statistics for Windows, version 28.0 was used for data analysis.

## Results

### Baseline characteristics

During the study period, 24 patients fulfilled the inclusion criteria and were included in the study cohort. In 22 out of these 24 patients, the underlying reason for respiratory hypercapnia was COVID-19-related ARDS. The other two non-COVID patients suffered from ARDS on top of necrotizing pancreatitis. 75% of the patients were male with a median age of 61 years and a median SOFA score of 15 at time of inclusion. Comorbidities mostly involved cardiovascular disease (58% of patients), while hypertension and diabetes were also frequent (Table 1).

**Table 1:** Baseline characteristics of the study participants. Data are shown as median (IQR) or number (%) as convenient

Characteristics	All (*n* = 24)
Age	61 (56–74)
Sex	
Female	6 (25%)
Male	18 (75%)
Weight (kg)	80 (72–90)
Height (cm)	175 (169–180)
BMI	27 (23–29)
Overweight (BMI ≥ 25):	15 (62.5%)
Obesity (BMI ≥ 30):	4 (16.7%)
Scores	
SOFA	15 (14–17)
GCS	3 (3–3)
Cause of hypercapnia	
ARDS	24 (100%)
Etiology of multiple organ failure	
COVID-19 ARDS	22 (92%)
Necrotizing pancreatitis	2 (8%)
Comorbidities	
Diabetes	6 (25%)
Cardiovascular disease	14 (58%)
Hypertension	9 (38%)
Chronic kidney disease	4 (17%)
Acute kidney injury	4 (17%)
Liver dysfunction	2 (8%)
Neoplasia	3 (13%)
Pancreatitis	2 (9%)
Sepsis	5 (21%)
Prior dialysis performed (> 2x/week)	13 (54%)
CVVHD	4 (17%)
IHD	8 (33%)
CVVHD + IHD	1 (4%)
Mechanical ventilation	24 (100%)
Days from intubation to 1st ADVOS treatment	13 (5, 19)
Total days of intubation	24 (17, 31)
Intensive Care Unit—Length of stay (ICU–LOS days)	24 (18, 37)
Days from ICU admission to 1st ADVOS treatment	13 (3, 19)

### ADVOS settings

A total of 134 ADVOS multi sessions were performed, with a median of 5 sessions per patient. Standard treatment parameters included anticoagulation with citrate and calcium, a median blood flow of 300 mL/min, a median concentrate flow of 320 mL/min and a median dialysate pH of 8.5. These data were obtained from the 458 timepoints documented along the 134 treatments. If required, settings were changed according to the manufacturer’s instructions. These include changes in the dialysate pH, the alkaline concentrates or the concentrate flow in reaction to patients changes according to BGA values. In these case series only 3 patients required blood flows higher than 300 mL/min and none of them within the first 24 h of ADVOS therapy.

ADVOS treatment settings are shown in detail in Table 2.

**Table 2:** ADVOS treatment settings

Treatment setting:	All (*n* = 24)
ADVOS treatments	
Total number of treatments	134
ADVOS treatments per patient	5 (3–8)
Treatment duration first 24 h (h)	22 (19–24)
General Treatment duration (h)	103 (53–184)
ADVOS settings (first 24 h)	
Blood flow max. (mL/min)	300 (300–300)
Dialysate pH	8.5 (8.3–8.6)
Concentrate flow (mL/min)	320 (240–320)
Ultrafiltration rate (mL/h)	13 (0–50)
Anticoagulation (first 24 h)	
No anticoagulation	0 (0%)
Citrate (alone)	1 (4%)
* Citrate rate (mL/h)*	218
* Calcium rate (mL/h)*	11.5
* Ionized calcium pre-dialyzer (mmol/L)*	1.07 (1.02–1.11)
UFH (alone)	0
Citrate and UFH	23 (96%)
* Citrate rate (mL/h)*	373 (334–452)
* Calcium rate (mL/h)*	20 (16–24)
* Ionized calcium pre-dialyzer (mmol/L)*	1.19 (1.11–1.28)
* Heparin dose (IU/h)*	952 (538–1491)
Number of discontinued treatments (first 24 h)	2 (8%)
Malfunctioning	1 (4%)
Clotting	1 (4%)
Duration of aborted treatments (h)	14 (13, 15)
Number of discontinued treatments (all 134 treatments)	28 (21%)
Malfunctioning	13 (9.7%)
Clotting	6 (4.5%)
Prone positioning	1 (0.7%)
Death/Last line/limitation of therapy	8 (6.0%)
Duration of aborted treatments (h)	16 (11–19)

Data are shown as median (IQR) or number (%) as convenient

### Time to correction of acid–base balance

**Fig. 1:**
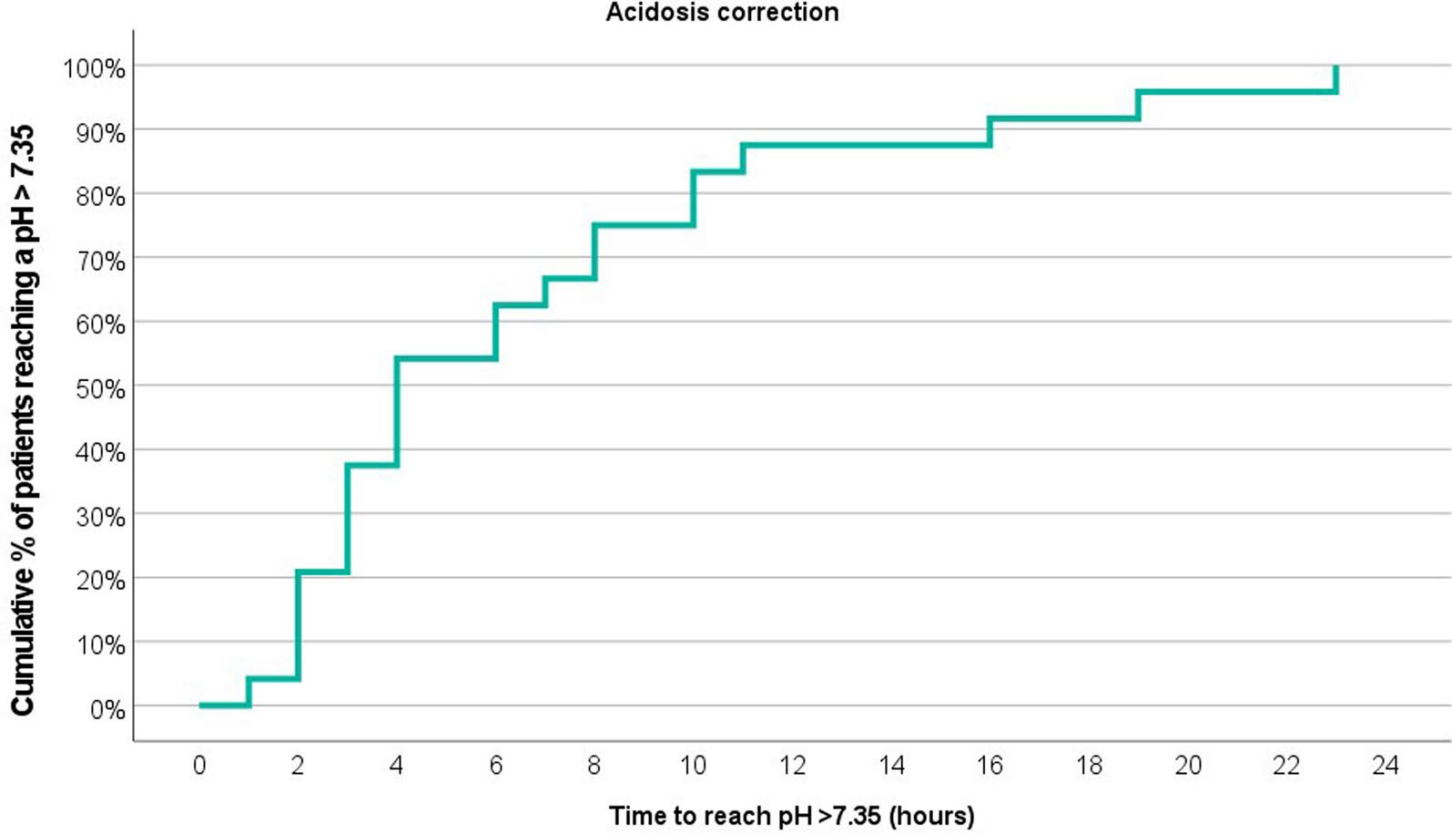
Kaplan–Meier curve for the cumulative % of patients reaching a blood pH ≥ 7.35 for the first time during the first 24 h of ADVOS treatment. Baseline median blood pH = 7.21

**Fig. 2:**
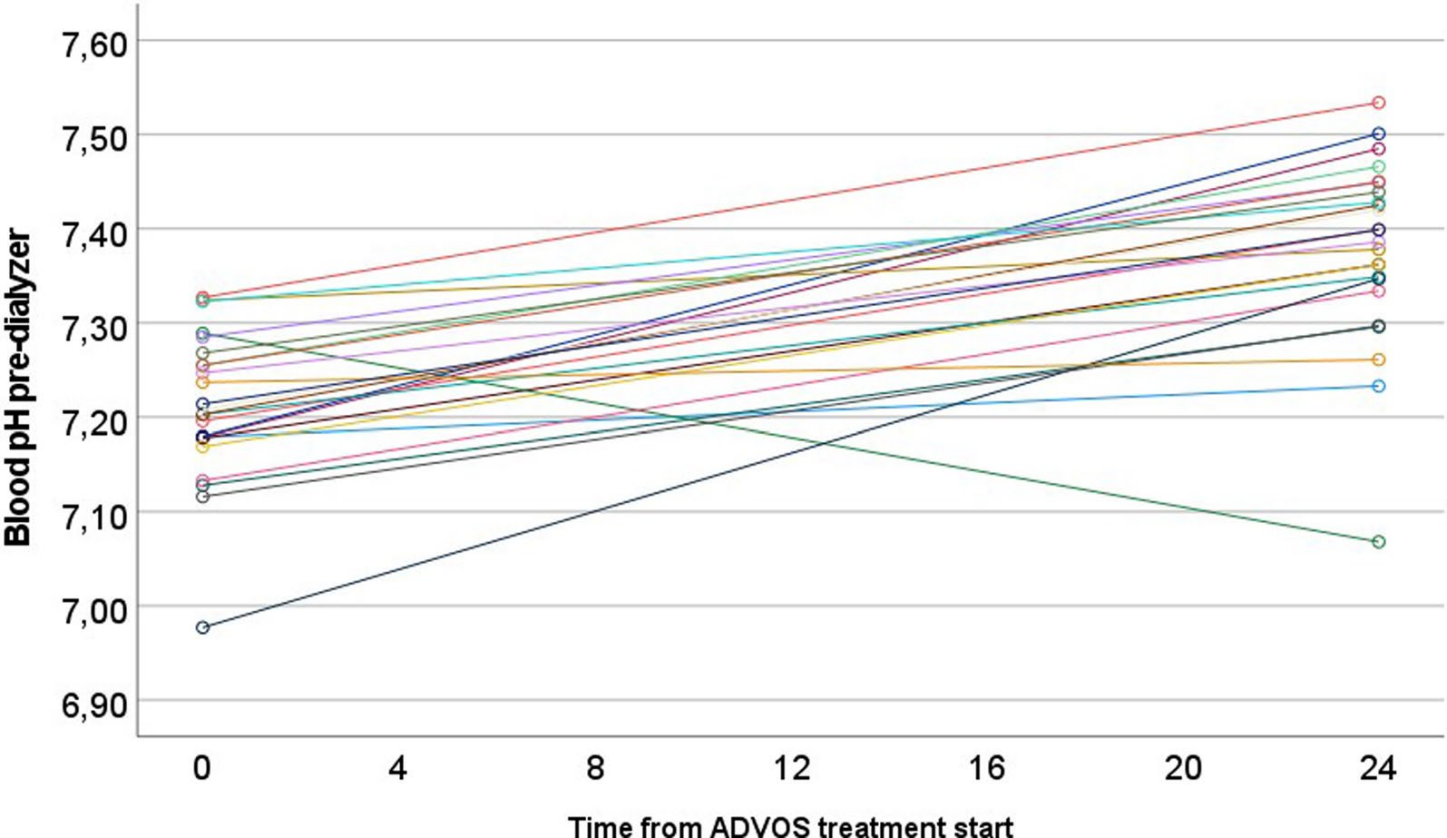
Change of blood pH from baseline to after 1st ADVOS treatment in each individual participant

ADVOS multi was able to restore hypercapnic acidosis to blood pH ≥ 7.35 in all the patients within a median of 4.3 h (IQR: 3.0–8.5 h) (Fig. 1; Table 3). Within 24 h, blood pH increased from 7.21 to 7.39 (*p* < 0.01). After the first 24 h of ADVOS treatment 18 out of 24 patients (75%) maintained blood pH values ≥ 7.35 (Fig. 2).

**Table 3:** Progress of performance parameters between baseline (0 h) and after the first ADVOS treatment (24 h)

	ALL (*n* = 24)			
	0 h	24 h	Paired differences	*p*
Blood pH	7.21 [7.18–7.26]	7.39 [7.34–7.45]	0.17 (0.12–0.21)	< 0.001 (*)
pCO_2_ (mmHg)	64 [52–74]	55 [49–69]	− 4.7 (− 12.8 to 3.4)	0.026 (*)
HCO_3_ (mmol/L)	24.3 [21.3–30.6]	34.5 [30.3–38.5]	7.7 (4.5–10.8)	< 0.001 (*)
Base Excess (mmol/L)	− 3 [− 6.6 to 2.3]	8.2 [3.3–12.7]	9.1 (6.2–12.1)	< 0.001 (*)
Sodium (mmol(L)	137 [133–142]	140 [136–141]	1.1 (− 1.3 to 3.6)	0.250
Chloride (mmol/L)	106 [102–112]	102 [101–103]	− 4.8 (− 7.4 to − 2.2)	< 0.001 (*)
Lactate (mmol/L)	1.4 [0.9–2]	1.2 [0.9–2]	0.1 (− 0.7 to 0.8)	0.896
SID (mEq/L)	37 [32–40]	41 [39–45]	5.4 (3.1–7.6)	< 0.001 (*)
Creatinine (mg/dL)	1.2 [0.7–1.7]	0.6 [0.4–1.1]	− 0.6 (− 0.8 to − 0.4)	< 0.001 (*)
BUN (mg/dL)	33 [21–50]	16 [10–23]	− 18.4 (− 24.3 to − 12.6)	< 0.001 (*)
Bilirubin (mg/dL)	0.5 [0.4–1.95]	0.85 [0.4–3.25]	0.04 (− 0.26 to 0.34)	0.658
CRP (mg/dL)	14.7 [7.9–21.9]	13.3 [9.4–18.2]	− 1.3 (− 4.1 to 1.6)	0.364
Procalcitonin (ng/mL)	1.2 [0.4–2.6]	0.8 [0.4–4.2]	6.1 (− 6.0 to 18.2)	0.537
Platelets (/nL)	167 [125–235]	135 [88–206]	− 30.7 (− 44.9 to − 16.4)	< 0.001 (*)
Noradrenaline (ug/kg/min)	0.260 [0–0.3]	0.05 [0–0.135]	− 0.070 (− 0.211 to 0.070)	0.074
Minute ventilation (L/min)	11.1 [10–12.1]	9.6 [9.1–11.7]	− 0.4 (− 1.1 to 0.4)	0.308
Tidal Volume (ml/kg)	5.3 [4.6–6.4]	4.8 [4.4–5.8]	0.1 (− 0.6 to 0.7)	0.861

Data show the very first treatment of each of the 24 patients, including those prematurely terminated. Median (IQR). Paired differences are shown as mean and lower and upper limits. (*) indicating statistical significance

*pCO*_*2*_ partial carbon dioxide, *HCO*_*3*_ bicarbonate, *SID* strong ion difference, *BUN* blood urea nitrogen, *CRP* C-reactive protein

To note, one patient deviated markedly from the rest, with what seems a paradoxical or delayed response. In this special case, the patient reached the goal after 4 h (pH = 7.382, pCO2 = 47.4 mmHg) but discontinued the treatment after 11 h due to a clotting issue before the dialyzer.

### CO2 removal rate and influence of ADVOS multi operational settings

The median CO_2_ removal rate during ADVOS treatments was 55 mL/min. In bivariate Pearson analyses and a multiple linear regression model, ADVOS multi operational settings and patient baseline levels showed statistically significant correlations with CO_2_ removal (Tables 4 and 5). Parameters included blood flow, concentrate flow, type of alkaline concentrate (i.e., bicarbonate content in dialysate), dialysate pH above 8.5, baseline pCO_2_ and baseline serum bicarbonate levels.

**Table 4:** Pearson correlation of ADVOS settings and baseline pCO_2_ and HCO_3_^−^ with CO_2_ removal

Parameters	Pearson correlation	n	*t* test	Significance (two-tailed)
Blood flow	0.649	458	18.198	< 0.001
Concentrate flow	0.145	458	3.136	0.004
Alkaline concentrate	− 0.477	458	− 11.584	< 0.001
Dialysate pH > = 8.5	0.397	458	9.234	< 0.001
pCO2 (mmHg)	0.299	458	6.700	< 0.001
HCO3 (mmol/L)	0.377	458	8.684	< 0.001

**Table 5:** Multiple linear regression model for CO_2_ removal predictors

	Coefficients	Standard error	t Stat	*p*	Lower 95%	Upper 95%
Intercept	− 40.175	10.309	− 3.897	0.000	− 60.435	− 19.915
Blood flow	0.207	0.017	12.441	0.000	0.174	0.240
Concentrate flow	− 0.023	0.019	− 1.224	0.222	− 0.060	0.014
Alkaline concentrate	− 0.804	0.155	− 5.197	0.000	− 1.108	− 0.500
Dialysate pH > = 8.5	3.868	2.853	1.356	0.176	− 1.739	9.475
pCO2 (mmHg)	0.366	0.084	4.335	0.000	0.200	0.532
HCO3 (mmol/L)	0.950	0.278	3.419	0.001	0.404	1.496

The current model shows an *R*^2^ of 0.532 and an F-statistic of 85.38, showing and overall significance (*p* < 0.001)

The highest CO_2_ removal was observed with a combination of high blood flow and an alkaline concentrate without bicarbonate (BASE Bic 0) at a dialysate pH above 8.5. Under these conditions, mean CO_2_ removal reached 76 ± 12 mL/min at 300 mL/min blood flow and up to 98 ± 31 mL/min at 350 mL/min (Table 6).

**Table 6:** CO_2_ removal (mL/min) for different combinations of blood flow, dialysate pH and alkaline concentrate during ADVOS multi treatments

	Dialysate pH	< 8.5			≥ 8.5		
	Alkaline concentrate	BASE BIC 20	BASE BIC 10	BASE BIC 0	BASE BIC 20	BASE BIC 10	BASE BIC 0
Blood flow							
100 mL/min		14 ± 10	16 ± 8	n.a	15 ± 9	24 ± 13	n.a
200 mL/min		30 ± 20	43 ± 18	n.a	26 ± 24	44 ± 16	46 ± 4
300 mL/min		44 ± 22	59 ± 25	n.a	64 ± 32	68 ± 23	76 ± 12
350 mL/min		n.a	n.a	n.a	n.a	73 ± 21	98 ± 31

Mean ± S.D. Combinations with less than five measurements are not shown (n.a.)

In the multiple linear regression model (Table 5), blood flow (*β* = 0.207, *p* < 0.001), baseline pCO₂ (*β* = 0.366, *p* < 0.001), baseline serum bicarbonate (*β* = 0.950, *p* = 0.001), and the use of BASE Bic 0 (*β* = − 0.804, *p* < 0.001) were independently associated with higher CO₂ removal. The model explained 53.2% of the variance in CO₂ elimination (adjusted *R*^2^ = 0.532, *F* = 85.38, *p* < 0.001).

**Fig. 3:**
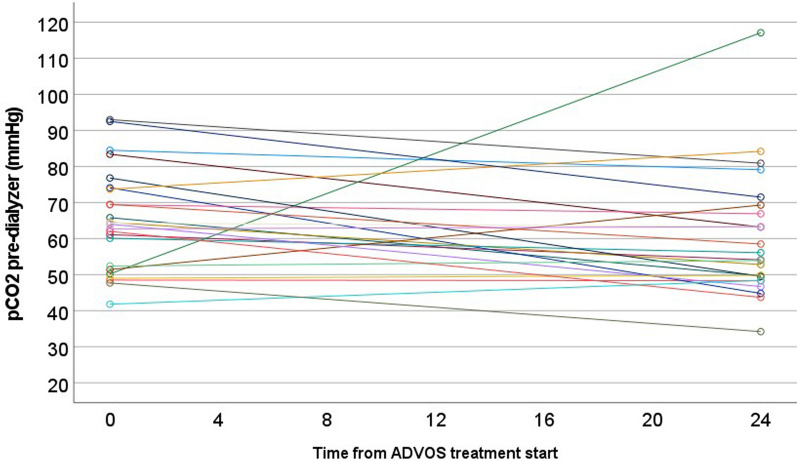
Change of pCO_2_ from baseline to after 1st ADVOS treatment in each individual participant

On top of CO_2_ elimination, pCO_2_ decreased significantly (64 vs. 55 mmHg, *p* = 0.026) after the first ADVOS session (Fig. 3).

To further evaluate the effect on gas exchange and metabolic acid removal, additional models were built using pCO₂ reduction (ΔpCO₂) and total CO₂ removal (i.e., combined pCO₂ and bicarbonate clearance) within the inlet and the outlet of the dialyzers as outcome variables (Supplementary Tables 1–4). For ΔpCO₂, baseline pCO₂ was the strongest predictor (*β* = 0.758, *p* < 0.001), while blood flow and concentrate flow were not statistically significant. For total CO₂ removal, blood flow, baseline HCO₃⁻, and the use of BASE Bic 0 remained significant predictors, with an adjusted *R*^2^ of 0.566 (*p* < 0.001).

The course of other treatment parameters is shown in Table 3.

## Discussion

### Key results

As mentioned above, acidosis is associated with increased in-hospital mortality, increased length of stay in the ICU, and prolonged hospital stay [1, 2]. Thus, severe hypercapnia especially when associated with acidosis should be actively managed.

Our findings demonstrate that the ADVOS multi hemodialysis system effectively corrects hypercapnic acidosis, achieving a median CO_2_ removal rate of 55 mL/min and blood pH normalization within 4 h. The analysis of factors influencing CO₂ removal revealed that both operational settings and patient-specific variables play a significant role. In bivariate correlation (Table 4), blood flow was most strongly associated with CO₂ elimination (*r* = 0.649, *p* < 0.001), followed by dialysate pH ≥ 8.5, baseline pCO₂, and the use of BASE Bic 0. In the multivariable model (Table 5), blood flow, baseline pCO₂, serum bicarbonate, and BASE Bic 0 were independently associated with CO₂ removal, with the model explaining 53.2% of the variance. Stratified analysis (Table 6) confirmed that the highest CO₂ clearance (up to 98 ± 31 mL/min) was achieved at blood flows ≥ 300 mL/min, with BASE Bic 0 and dialysate pH ≥ 8.5. To better reflect clinical relevance, we, therefore, evaluated pCO₂ reduction (ΔpCO₂) and total CO₂ removal (including bicarbonate elimination) in additional models (Supplementary Tables 1–4). While blood flow lost significance for ΔpCO₂, baseline pCO₂ remained the dominant predictor (*β* = 0.758, *p* < 0.001), and BASE Bic 0 combined with pH ≥ 8.5 continued to show favorable effects. In the model for total CO₂ removal, baseline HCO₃⁻ and BASE Bic 0 were again strong and independent predictors. Together, these results clarify that while mathematical CO₂ removal is influenced by flow and gradient components, the clinically meaningful impact on acid–base status depends on a combination of patient factors (baseline pCO₂ and HCO₃⁻) and dialysate composition, particularly alkaline concentrate type and pH. This supports the physiological and clinical rationale for customizing ADVOS settings to optimize both ventilatory support and metabolic acid–base correction in patients with hypercapnic acidosis.

### Interpretation and generalizability

These findings underline the potential of the ADVOS multi hemodialysis system as an adjunctive therapy for managing hypercapnic acidosis in critically ill patients, especially in in patients with multi organ failure when conventional therapies, such as mechanical ventilation, are insufficient [18].

A previous study with a lower number of patients with acidosis showed that blood pH correction could be achieved within 6 h [15], which could be confirmed in this study. This is in line with a larger study by Jung et al. in which the rapidity of acidosis correction appeared to be a more important determinant of clinical outcome rather than the initial blood pH value itself [19].

Based on the pH optimization through dialysate pH adjustment and bicarbonate content capabilities of the ADVOS multi system, a precise acid–base correction and CO_2_ removal at low blood flow level (100–400 mL/min), could be a safe alternative if ECMO or ECCO_2_R is not possible as demonstrated in this study [20].

ECMO as well as ECCO_2_R therapy left some controversial discussions in the treatment of critically ill patients. In particular, the REST trial assessed the role of ECCO₂R in patients with acute hypoxemic respiratory failure and found no significant reduction in 90-day mortality [5]. Moreover, ECCO₂R was associated with a higher rate of serious adverse events, including bleeding complications. These findings raise concerns about the widespread adoption of ECCO₂R as a routine intervention. In contrast, ADVOS achieves CO₂ removal through a fluid-based approach rather than direct gas exchange, potentially offering a safer alternative with a lower risk of anticoagulation-related complications as reported in this study.

As an alternative, the use of bicarbonate therapy for metabolic acidosis has been debated. The BICAR–ICU trial found that sodium bicarbonate infusion did not significantly improve 28-day survival in the general ICU population with metabolic acidosis, but it did reduce mortality and renal replacement therapy (RRT) requirements in patients with acute kidney injury (AKI) [21]. The ongoing BICARICU-2 trial is specifically evaluating whether bicarbonate therapy improves 90-day mortality in patients with both severe metabolic acidemia and AKI [22]. In contrast to conventional bicarbonate therapy, as presented above, ADVOS enables a more controlled and targeted rapid correction of acidosis without the risk of metabolic alkalosis, hypernatremia, or intracellular CO₂ accumulation. By continuously modulating dialysate pH and bicarbonate content, ADVOS offers a dynamic and patient-specific approach to acid–base management, which may be advantageous in critically ill patients with complex acid–base disturbances.

In any case, neither the REST nor the BICAR–ICU trials assessed the timing to pH correction with ECCO_2_R or bicarbonate therapy.

A more recent strategy involves CO₂-adapted kidney replacement therapy (KRT), as explored in the BigBIC trial, which tailored bicarbonate levels to physiological renal compensation in hypercapnic ARDS patients requiring dialysis [23]. This approach increased systemic bicarbonate levels, facilitated lung-protective ventilation, and showed a trend toward reduced 30-day mortality (42% vs. 63%), though statistical significance was not reached [23]. While both ADVOS and CO₂-adapted KRT modify dialysis settings to optimize acid–base balance, ADVOS provides greater precision in dialysate composition, allowing for more efficient CO₂ removal while simultaneously addressing toxin elimination via albumin-based filtration.

## Limitations

Several limitations must be acknowledged. First, this was an observational single-center study with a small sample size (*n* = 24) and the absence of a control group, limiting statistical power and generalizability. However, the conclusions are supported by data from 458 blood gas measurements, which strengthens the robustness of the findings. Second, while this study assessed short-term pH correction, long-term outcomes, such as ICU ventilator dependency, and survival rates, were not evaluated. Moreover, it could have been interesting to stratify pure hypercapnic patients and those with mixed acidosis. Ongoing clinical trials are already using a randomized and controlled design and will analyze different acidosis types. Third, the study was conducted in a single center, which may introduce biases related to specific institutional practices and patient populations. Fourth, the reliance on pre- and post-filter blood gas analyses provides insight into immediate changes but may not fully capture systemic effects of ADVOS therapy. Fifth, the paradoxical course of one patient whose pH normalized after 4 h of treatment, but who developed acidosis after completion of the treatment can be explained by the fact that treatment had to be discontinued after 11 h due to a clotting issue. Finally, 21% of the treatments had to be discontinued before 24 h due to common reasons depicted in Table 2. This might limit the validity of the results. However, only 2 were prematurely stopped after a median duration of 14 h within the first 24 h treatments in each patient, which does not affect the conclusion of the work.

## Future prospects

The ability of ADVOS to correct both metabolic and respiratory acidosis in a tailored manner makes it particularly relevant for patients with multiple organ failure, where mixed acid–base disorders are common. The precise titration of bicarbonate levels in the dialysate, combined with its albumin-based detoxification capacity, provides a broader physiological impact compared to ECCO_2_R, bicarbonate therapy or traditional renal replacement therapies.

However, further research is needed to establish the long-term benefits of ADVOS on clinical outcomes, including ICU length of stay, ventilator-free days, and survival. Large, multicenter trials similar to those conducted for bicarbonate therapy and ECCO₂R would help define its optimal use and comparative effectiveness in different patient populations.

Indeed, at the time of writing this report, several clinical trials for the improvement of different clinical outcomes with ADVOS were ongoing (e.g., DRKS00031279 and NCT05842369). Finally, the development of optimized protocols for ADVOS settings, including blood flow and bicarbonate content, could further enhance its efficacy and safety.

## Conclusion

The ADVOS multi hemodialysis system effectively normalized blood pH within a median of 4.3 h by facilitating CO_2_ removal at a median rate of 55 mL/min in patients with multiple organ failure and hypercapnic acidosis. Main determinants for CO_2_ removal were high blood flows and the use of bicarbonate-free concentrates, especially if combined with a high dialysate pH above 8.5. Future studies should further investigate its impact on mechanical ventilation and circulatory support.

## Supplementary Information

Supplementary material 1.

## Data Availability

The data sets used and/or analyzed during the current study are available from the corresponding author on reasonable request.

## Abbreviations

ADVOS: ADVanced Organ Support
AKI: Acute kidney injury
ARDS: Acute respiratory distress syndrome
BMI: Body mass index
ECCO2R: Extracorporeal carbon dioxide removal
ECMO: Extracorporeal membrane oxygenation
RRT: Renal replacement therapy
