# Supplementary material for: The ADVanced Organ Support (ADVOS) hemodialysis system balances blood pH within 24 h in patients with multiple organ failure and hypercapnic acidosis
Source: Intensive Care Med Exp. 2025 Oct 25;13:108. doi: 10.1186/s40635-025-00820-1 (PMC12553777; doi:10.1186/s40635-025-00820-1)
Supplement: Supplementary file 1 — Supplementary material 1. [file 40635_2025_820_MOESM1_ESM.docx]

**Supplementary Information**

**Supplementary Table 1**. Pearson correlation of ADVOS settings and baseline pCO_2_ and HCO_3_^-^ with pCO_2_ reduction between the inlet and the outlet of the dialyzer.

| **Parameters** | **Pearson Correlation** | **n** | **t-test** | **Significance (2-tailed)** |
| --- | --- | --- | --- | --- |
| Blood flow | 0,156 | 458 | 3.311 | 0.002 |
| Concentrate flow | 0,194 | 458 | 4.224 | <0.001 |
| Alkaline concentrate | -0,242 | 458 | -5.277 | <0.001 |
| Dialysate pH >=8,5 | 0,370 | 458 | 8.480 | <0.001 |
| pCO2 (mmHg) | 0,805 | 458 | 29.097 | <0.001 |
| HCO3 (mmol/L) | 0,286 | 458 | 6.358 | <0.001 |

**Supplementary Table 2**. Multiple linear regression model for pCO2 reduction predictors. The current model shows an R^2^ of 0.669 and an F-statistic of 151.65, showing an overall significance (p < 0.001).

|  | *Coefficients* | *Standard Error* | *t Stat* | *p* | *Lower 95%* | *Upper 95%* |
| --- | --- | --- | --- | --- | --- | --- |
| Intercept | -14.291 | 3.741 | -3.820 | <0.001 | -21.642 | -6.939 |
| Blood flow | -0.001 | 0.006 | -0.126 | 0.899 | -0.013 | 0.011 |
| Concentrate flow | 0.008 | 0.007 | 1.167 | 0.244 | -0.050 | 0.022 |
| Alkaline concentrate | -0.109 | 0.056 | -1.946 | 0.052 | -0.220 | 0.001 |
| Dialysate pH >=8,5 | 3.179 | 1.035 | 3.071 | 0.002 | 1.145 | 5.214 |
| pCO2 (mmHg) | 0.758 | 0.031 | 24.763 | <0.001 | 0.698 | 0.818 |
| HCO3 (mmol/L) | 0.021 | 0.101 | 0.208 | 0.836 | -0.177 | 0.219 |

**Supplementary Table 3**. Pearson correlation of ADVOS settings and baseline pCO_2_ and HCO_3_^-^ with total CO_2_ reduction (pCO_2_ + HCO_3_^-^) between the inlet and the outlet of the dialyzer.

| **Parameters** | **Pearson Correlation** | **n** | **t-test** | **Significance (2-tailed)** |
| --- | --- | --- | --- | --- |
| Blood flow | 0,156 | 458 | 3.311 | 0.002 |
| Concentrate flow | 0,194 | 458 | 4.224 | <0.001 |
| Alkaline concentrate | -0,242 | 458 | -5.277 | <0.001 |
| Dialysate pH >=8,5 | 0,370 | 458 | 8.480 | <0.001 |
| pCO2 (mmHg) | 0,805 | 458 | 29.097 | <0.001 |
| HCO3 (mmol/L) | 0,286 | 458 | 6.358 | <0.001 |

**Supplementary Table 4**. Multiple linear regression model for total CO_2_ reduction (pCO_2_ + HCO_3_^-^) reduction predictors. The current model shows an R^2^ of 0.296 and an F-statistic of 151.65, showing and overall significance (p < 0.001).

|  | *Coefficients* | *Standard Error* | *t Stat* | *p* | *Lower 95%* | *Upper 95%* |
| --- | --- | --- | --- | --- | --- | --- |
| Intercept | -14.291 | 3.741 | -3.820 | <0.001 | -21.642 | -6.939 |
| Blood flow | -0.001 | 0.006 | -0.126 | 0.899 | -0.013 | 0.011 |
| Concentrate flow | 0.008 | 0.007 | 1.167 | 0.244 | -0.050 | 0.022 |
| Alkaline concentrate | -0.109 | 0.056 | -1.946 | 0.052 | -0.220 | 0.001 |
| Dialysate pH >=8,5 | 3.179 | 1.035 | 3.071 | 0.002 | 1.145 | 5.214 |
| pCO2 (mmHg) | 0.758 | 0.031 | 24.763 | <0.001 | 0.698 | 0.818 |
| HCO3 (mmol/L) | 0.021 | 0.101 | 0.208 | 0.836 | -0.177 | 0.219 |
